# Lower cognitive scores among toddlers in birth cohorts with acute respiratory illnesses, fevers, and laboratory‐confirmed influenza

**DOI:** 10.1111/irv.12904

**Published:** 2021-09-14

**Authors:** Eduardo Azziz‐Baumgartner, Rosalba Gonzalez, William Davis, Arlene Calvo, Natalie Olson, Lauren Grant, Morgan Hess‐Holtz, Vic Veguilla, Rafael Rauda, Susan Cornelia Kaydos‐Daniels, Nestor Sosa, Evelina I. Aedo Ruíz, Julio Armero Guardado, Rachael Porter, Danilo Franco, Juan Miguel Pascale, Georgina Peacock

**Affiliations:** ^1^ Centers for Disease Control and Prevention Atlanta GA USA; ^2^ Gorgas Institute Panama City Panama; ^3^ University of South Florida Tampa Florida USA; ^4^ National Institute of Health of El Salvador San Salvador El Salvador; ^5^ CICAP Panama City Panama

**Keywords:** acute respiratory illness, cognitive development, febrile illness, influenza

## Abstract

**Background:**

We established cohorts to assess associations between viral influenza and cognitive development to inform the value proposition of vaccination.

**Methods:**

From 2014 through 2017, we called women seeking care at four prenatal clinics in Panama and El Salvador to identify acute respiratory illnesses (ARIs). Within 2 weeks of childbirth, mothers were asked to enroll their neonates in the cognitive development study. Staff obtained nasopharyngeal swabs from children with febrile ARIs for real‐time reverse transcription polymerase chain reaction (rtPCR) detection of viral RNA. Toddlers were administered Bayley developmental tests at ages 12 and 18–24 months. We used multilevel linear regression to explore associations between Bayley scores, ARIs, fever, and laboratory‐confirmed influenza, controlling for maternal respiratory or Zika illnesses, infant influenza vaccination, birth during influenza epidemics, and the number of children in households.

**Results:**

We enrolled 1567 neonates of which 68% (n = 1062) underwent developmental testing once and 40% (*n* = 623) twice. Children with previous ARIs scored an average of 3 points lower on their cognitive scores than children without ARIs (*p* = 0.001). Children with previous fevers scored an average of 2.1 points lower on their cognitive scores than afebrile children (*p* = 0.02). In the second year, children with previous laboratory‐confirmed influenza scored 4 points lower on their cognitive scores than children without influenza (*p* = 0.04, after controlling for first Bayley cognitive scores).

**Conclusions:**

ARIs and fever during infancy were associated with lower Bayley scores at 12 months, and laboratory‐confirmed influenza was associated with lower cognitive scores at 24 months suggesting the potential value of vaccination to prevent non‐respiratory complications of influenza.

**ABBREVIATIONS**
ARI: acute respiratory illness
CDC: Centers for Disease Control and Prevention
IQR: interquartile range
rtPCR: real‐time reverse transcription polymerase chain reaction
TORCH: Toxoplasmosis, Other Agents, Rubella, Cytomegalovirus, and Herpes simplex

## 1 INTRODUCTION

While influenza vaccination cost‐effectiveness research typically focuses on the prevention of acute respiratory illnesses (ARIs),^1^ influenza is also associated with acute and subacute neurologic sequelae^2^ that may influence vaccination's value proposition. During the 2009 pandemic, 1/5 persons with influenza admitted to intensive care units had neurological manifestations.^3^ Neurologic complications typically occurred within 5 days from symptom onset and disproportionally among racial and ethnic minorities and children.^4^, ^5^ More than 20% of hospitalized young children with influenza had febrile seizures, compared with 2% to 5% of other children, double that of parainfluenza or adenovirus.^6^

Pathogenesis of acute influenza‐associated neurological complications is not well understood and may include temporary and/or subacute neurological changes precipitated by cytokine and immune dysregulation.^2^, ^7^, ^8^ Mouse models suggest that influenza A virus illnesses may also be associated with long‐term neuroinflammation and cognitive deficits.^9^ Influenza infections in mice have been associated with recruitment and upregulation of astrocytes; dendritic blunting; remodeling of the hypothalamus responsible for motivation, emotion, learning, and memory; and poor performance on maze challenges. In other mouse models, neonatal influenza infection led to a proinflammatory cytokine and neural dysfunction.^10^ Although such mouse models suggest proinflammatory changes were associated with acute, subacute, and long‐term changes in rodent brains, the implications to humans remain unclear.

The few existing human birth cohort studies suggest febrile respiratory illnesses are associated with subacute changes in brain function.^11^ A birth cohort in Bangladesh, for example, found an association between days of febrile illness, proinflammatory cytokines, and lower language and motor development scores during infancy,^12^ but findings are difficult to interpret because of limited study duration and insufficient adjustment for factors associated with cognitive development.^13^ It is unclear if studies that could better account for maternal and infancy factors would still find associations between febrile respiratory illnesses and delayed cognitive development. Even if such findings were replicable, it is unclear whether cognitive development would be specifically associated with vaccine preventable influenza. We believe it is important to identify potential associations between influenza and cognitive development because; if these exist, this might strengthen the value proposition of influenza vaccination among infants aged ≥6 months to prevent such cognitive delays in settings where respiratory illnesses alone seem insufficiently compelling to providers and parents to achieve high vaccination coverage.^14^ We therefore established a cohort in Panama and El Salvador, at sites where we had completed an oseltamivir clinical trial among young children,^15^ to test whether delays in cognitive development during the first 2 years of life are associated with (1) febrile respiratory illnesses and (2) specifically laboratory‐confirmed influenza; both countries had demonstrated substantive influenza illness among young children.^16^

## 2 METHODS

Women seeking prenatal care in their first or second trimester at two government‐subsidized periurban clinics in Panama and two in El Salvador were contacted for enrollment. Eligible mothers were 16 years or older and signed informed consent for their neonates' participation. Those who consented were surveyed about their demographics, prenatal history, and ultrasound‐estimated gestational age. They were contacted once a week during their pregnancy and screened for ARI symptoms (i.e., acute onset of fever, cough, sore throat, rhinitis, sneezing, and/or respiratory distress) and pregnancy complications. Staff obtained nasopharyngeal samples from mothers with febrile ARIs within 7 days of illness onset for respiratory virus polymerase chain reaction (PCR) testing. Staff also offered mothers participation in a Zika substudy where participants could provide blood and urine samples for Zika PCR within 14 days of fever, rash, or conjunctivitis onset; those with positive Zika PCR or IgM tests and negative dengue IgM were considered Zika cases.

Staff systematically recorded maternal vaccination, laboratory results, ultrasound dates throughout pregnancy, and birth outcomes. At birth, children were weighed and measured using calibrated infantometers. Within 2 weeks of childbirth, mothers were asked to enroll their neonates in the cognitive development study. Neonates with Toxoplasmosis, Other Agents, Rubella, Cytomegalovirus, and Herpes simplex (TORCH) syndrome, chromosomal abnormalities, or other congenital illnesses were ineligible for enrollment because of the difficulty of distinguishing the effects of these conditions from that of ARIs on infants' development. Enrolled neonates were followed once a week by phone calls or texts to identify ARIs (i.e., defined as ≥1 respiratory symptom such as cough, rhinorrhea, or difficulty breathing) or other acute illness symptoms (e.g., asthenia, anorexia, irritability, or vomiting) in the preceding week. Children with ARIs were tested for respiratory pathogens if they developed subjective or measured temperature ≥38°C. During routine preventive care visits, parents were also asked about acute illnesses their children might have had since their previous well‐child visit.

Staff asked mothers to bring infants with febrile ARIs to the clinic within 7 days of illness onset to obtain nasopharyngeal swabs. Real‐time reverse transcription PCR (rtPCR) singleplex testing was conducted to detect influenza viruses, respiratory syncytial virus, parainfluenza viruses 1–3, adenoviruses, human metapneumovirus, and rhinovirus ribonucleic acid using Centers for Disease Control and Prevention (CDC) testing protocols.^17^ Study staff systematically recorded children's laboratory results, imaging studies, and vaccines obtained through routine preventive and/or acute clinical care. Unscheduled visits to clinics or hospitals were also recorded. ARI episodes separated by more than 2 weeks without symptoms were considered separate episodes.

Trained study psychologists piloted and administered the Spanish version of the Pearson Bayley Scales of Infant and Toddler Development®|third edition^18^, ^19^ to toddlers on their first and second birthdays, which generated age‐adjusted composite percentile scores for cognitive, language (containing expressive and receptive scores), and motor development. A small proportion of children had their second Bayley III administered ≤6 months after their first Bayley because of the end of study funding, when children were aged approximately 18 months. All tests were administered in study clinics and were not accompanied by home visits because of resource constraints.

We used univariate (for the first Bayley score) and bivariate (for the second Bayley score, controlling for the first score) multilevel mixed effects regression models with individuals at the first level, clinics at the second level, and countries at the third level, to examine associations between cognitive Bayley test scores and maternal and infancy exposures frequently associated with development. Multilevel models were used to account for possible clustering by clinic and country. Maternal exposures included age, pre‐existing conditions (Table 2), substance abuse, education, monthly household income (as a binary variable of being the lowest income quintile—<$US400 per month income—or not),^13^ presence of fever or acute respiratory symptoms, laboratory‐confirmed influenza, respiratory syncytial virus, parainfluenza viruses 1–3, adenoviruses, human metapneumovirus, rhinovirus and Zika. Infant exposures included race/ethnicity, premature birth, born during influenza season (defined as birth during epidemic weeks when the proportion of samples testing positive for influenza at the national reference laboratories where above the annual mean for >2 consecutive epidemic weeks^20^), female sex, twin, weight, length and height for age, acute respiratory infection (as yes/no), total number of acute respiratory infections, hospitalization with bronchopneumonia, fever, influenza vaccination (none, partial, and full), and laboratory‐confirmed influenza. We assumed mothers who did not report fever or respiratory symptoms did not have respiratory illnesses. We also assumed children with ARIs who were untested for respiratory viruses would have tested negative for influenza if these illnesses occurred during epidemiologic weeks without any influenza detections through national surveillance at reference laboratories in each country.

We used stepwise selection to build multilevel mixed effects linear regression models with individuals at the first level, clinics at the second level, and countries at the third level, with Bayley cognitive scores at ages 1 or 2 as dependent variables; variables that had univariate or bivariate associations of *p* ≤ 0.1 were considered for the model. We also tested variables that have been previously reported to influence cognitive development or been associated with risk of respiratory illnesses: maternal Zika illness and respiratory virus illnesses (tested as individual and combined variables), infant influenza vaccination, number of household members, children, and children aged <5 years, regardless of their significance in univariate or bivariate regressions. We excluded variables associated with study retention, and we repeated the analysis excluding twins. All analyses were done in STATA 15 (College Station, TX: StataCorp LLC).

To assess potential bias from missing data because of loss to follow‐up, we used *t* tests and chi‐squared tests to examine associations between baseline variables and participants who dropped out of the study. Variables associated with dropout and their potential to introduce bias are discussed in limitations.

The study was funded through CDC cooperative agreement 5U01IP000791‐05. The protocol was reviewed and approved by the Panama and El Salvador Institutional Review Boards; CDC deferred to Panama and El Salvador ethical review.

## 3 RESULTS

### 3.1 Enrollment and analytic sample

**FIGURE 1:**
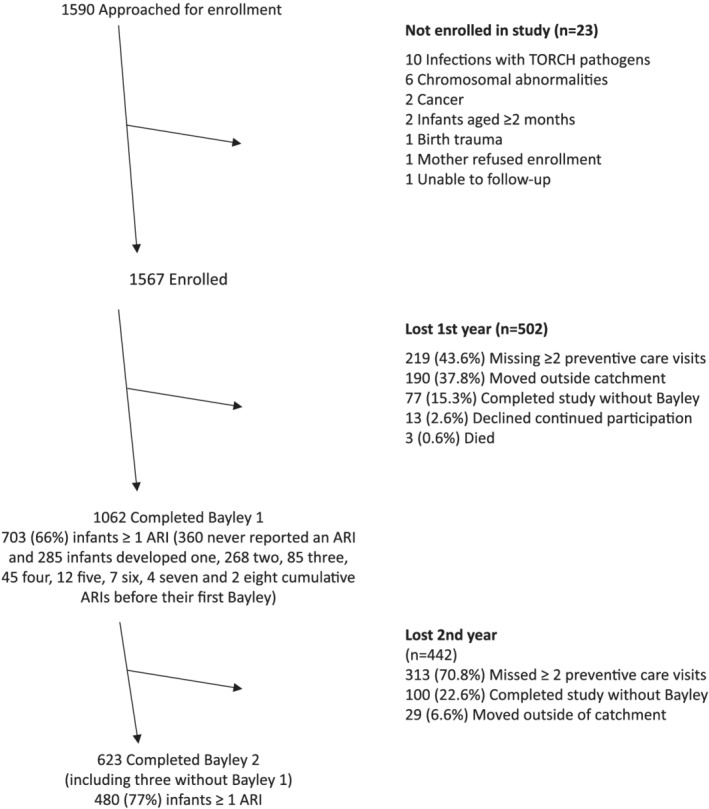
Enrollment of neonates into birth cohort to assess potential associations between acute respiratory illnesses (ARIs) and Bayley development scores, four clinics in Panama and El Salvador, December 2014 to November 2017

During December 29, 2014 to November 19, 2017, we identified 1590 neonates and enrolled 1567 (99%, Figure 1). Thirty‐two percent (502) of the 1567 enrolled neonates were lost to follow‐up during their first year of life. We tested cognitive development of the remaining 1062 (68%) during their first year of life; these became our primary analytic sample. An additional 458 (29%) were lost to follow‐up in the second year of life. During their second year of life, we retested the development of 620 (58%) of the 1062 children with a Bayley III in the first year plus an additional three children without a first‐year Bayley; these 623 became our secondary analytic sample. Children from Indigenous families were more likely to drop out before study completion (Table 1, *p* < 0.0001).

**TABLE 1:** Child retention in study by maternal and infant characteristics

	Enrolled but did not complete first year	Completed first year but not second year	Completed second year	Total	*p* if <0.05
Panama	387 (44%)	241 (28%)	243 (28%)	871	<0.0001
El Salvador	109 (16%)	217 (31%)	370 (53%)	696	
Maternal characteristics					
Median age	24	24	24		
Primigravid	151 (26%)	175 (30%)	252 (44%)	578	0.001
Indigenous	116 (51%)	64 (28%)	46 (20%)	226	<0.0001
Married	50 (26%)	50 (26%)	90 (47%)	190	0.04
Less than middle school	103 (31%)	94 (28%)	140 (42%)	337	
Intimate partner violence	32 (40%)	22 (27%)	27 (33%)	81	
Pre‐existing condition	47 (36%)	35 (27%)	49 (37%)	131	
Alcohol use	12 (44%)	8 (30%)	7 (26%)	27	
Smoker	4 (44%)	1 (11%)	4 (44%)	9	
Infant characteristics					
Female	157 (24%)	203 (31%)	291 (45%)	651	
Twins	4 (22%)	4 (22%)	10 (56%)	18	
Premature	29 (31%)	25 (27%)	40 (43%)	94	<0.0001
Weight *Z* score < −2	42 (30%)	40 (29%)	56 (41%)	138	

### 3.2 Maternal characteristics

Most mothers in El Salvador described themselves as Mestizo (586, 99.8%); mothers in Panama self‐identified as Mestizo (273, 56.4%), Indigenous (110, 22.7%), White (72, 14.9%), Black (27, 5.6%), or other (2, 0.4%) (Table 2). Mothers in El Salvador were more likely to have less than a seventh‐grade education (n = 158, 28%) and earn a household income of <$US400 a month (n = 450, 91%), the lowest income quintile, than mothers in Panama (n = 76, 16%; n = 126, 37% respectively; *p* < 0.001). Mothers in Panama, however, were more likely to report pre‐existing conditions (62, 13%) compared with mothers in El Salvador (22, 4%; *p* < 0.001). One in five mothers (n = 214, 20.1%) developed at least one acute respiratory illness during pregnancy of which nine (4%) tested positive for influenza. Only 432 (41%) of 1065 mothers participated in the Zika substudy, of whom 20 (4.6%) tested positive for Zika.

**TABLE 2:** Mother and child demographic characteristics and laboratory results by country

	Panama N = 480 (%)	El Salvador N = 585 (%)	*p* value	Overall N = 1065^a^ (%)
Maternal characteristics				
Age median (IQR)	23 (20–28)	22 (19–27)	0.0001	23 (19–28)
Primigravid	142 (29.6%)	284 (48.5%)	<0.001	426 (40.0%)
Indigenous	110 (22.9%)	0 (0%)	<0.001	110 (10.3%)
Mestizo	273 (56.4%)	586 (99.8%)	<0.001	859 (80.2%)
White	72 (14.8%)	0 (0%)	<0.001	72 (6.7%)
Black	27 (5.6%)	1 (0.2%)	<0.001	28 (2.6%)
Other	2 (0.4%)	0 (0%)	<0.001	2 (0.2%)
Married	37 (7.7%)	103 (17.6%)	<0.001	140 (13.1%)
Primary school or less	76 (15.8%)	158 (27.0%)	<0.001	234 (22.0%)
Intimate partner violence	43 (9.2%)	6 (1.1%)	<0.001	49 (4.8%)
Pre‐existing condition	62 (12.9%)	22 (3.8%)	<0.001	84 (7.9%)
Monthly household income < $US400 (lowest quintile)	126 (26.7%)	450 (76.9)	<0.001	576 (54.1%)
Drinks alcohol	14 (2.9%)	1 (0.2%)	<0.001	15 (1.4%)
Smoker	3 (0.6%)	2 (0.3%)	0.5	5 (0.5%)
Influenza vaccination^b^	230 (47.9%)	81 (13.9%)	<0.001	311 (29.2%)
Acute respiratory illness	96 (20%)	118 (20.2%)	1.0	214 (20.1%)
Influenza^c^	2 (0.4%)	7 (1.2%)	0.06	9 (0.8%)
Zika^d^	6 (2.8%)^d^	14 (6.5%)^d^	0.07	20 (4.6%)^d^
Infant characteristics				
Female	214 (44.6%)	278 (47.5%)	0.4	492 (46.2%)
Twins	6 (1.2%)	2 (0.3%)	0.2	8 (0.8%)
Gestational age in weeks (IQR)	39 (38–40)	39 (38–40)	<0.001	39 (38–40)
Premature	22 (4.6)	42 (7.2)	0.09	64 (6%)
Weight *Z* < −2^e^	14 (2.9%)	23 (3.9%)	0.4	37 (3.5%)
Length *Z* < −2^e^	8 (1.7%)	19 (3.2)	0.2	27 (2.5%)
Head circumference *Z* < −2^e^	21 (4.4%)	26 (4.4%)	1	47 (4.4%)
Any influenza vaccination	301 (62.7%)	128 (21.9%)	<0.001	429 (40.3%)
Fully vaccinated against influenza	156 (32.5%)	25 (4.3%)	<0.001	181 (17.0%)
Any ARI before first Bayley	361 (75.2%)	341 (58.3%)	<0.001	702 (65.9%)
Any ARI before second Bayley^f^	221 (75.2%)	269 (57.8%)	<0.001	490 (64.6%)
Feverishness^c^ during study	330 (68.9%)	328 (56.4%)	<0.001	658 (62.0%)
Median cumulative days of fever (IQR)	6 (4–11)	5 (3–8)	<0.001	6 (3–9)
Hospitalized	56 (11.7)	35 (6.0)	0.001	91 (8.5%)
Influenza^g^	29 (6.0%)	27 (4.6%)	0.001	52 (4.9%)
Respiratory syncytial virus^g^	48 (10%)	43 (7.4%)	0.01	91 (8.5%)
Adenovirus^g^	22 (8.3%)	17 (6.5%)	0.4	39 (7.4%)
Human metapneumovirus^g^	25 (5.2%)	35 (6.0%)	0.5	60 (5.6%)
Rhinovirus^g^	107 (22.3%)	123 (21.0%)	1.0	230 (21.6%)
Parainfluenza 1^g^	10 (2.1%)	15 (2.6%)	0.7	25 (2.3%)
Parainfluenza 2^g^	1 (0.2%)	7 (1.2%)	0.08	8 (0.8%)
Parainfluenza 3^g^	32 (6.7%)	22 (3.8%)	0.05	54 (5.1%)

Abbreviations: ARI, acute respiratory illnesses; CDC, Centers for Disease Control and Prevention; IQR, interquartile range; rtPCR, real‐time reverse transcription polymerase chain reaction.

^a^ Three children solely completed a Bayley during 2nd year of life (not a first‐year Bayley).

^b^ Vaccination with inactivated trivalent vaccine ≤1 year of child's 6‐month birthday.

^c^ Measured or subjective fever.

^d^ Solely 432 (41%) of 1065 participated in Zika substudy (217 in Panama ad 215 in El Salvador).

^e^ Adjusted for gestational age in weeks.

^f^ Among 623 children with second Bayley.

^g^ Number of mothers or children with viral RNA detections through rtPCR testing using CDC protocols.

### 3.3 Infant characteristics

Approximately half (492 [46%]) of the 1065 children were female (Table 2). Most (1006, 95%) were born at term; 65 (6%) were preterm (median 34.7 weeks, IQR 33.3–35.3). Mean birth weight was 3.1 kg (IQR 2.8–3.4 kg), and 37 (4%) had a birth weight ≥2 standard deviations lower than other cohort children after adjusting for gestational age. Children were followed for a median 12.1 months (IQR 12.1–12.4) before their first Bayley and 24.2 months (IQR 24.1–24.4) before their second Bayley. More infants aged 6–12 months were fully vaccinated against influenza in Panama (156, 32.5%) than in El Salvador (25, 4.3%, *p* < 0.001).

### 3.4 ARIs and laboratory results

Sixty‐six percent (703) of the 1062 infants with a first Bayley test developed ≥1 ARI before their test (cumulative 1402 ARIs, Figure 1). Seventy‐seven percent (480) of the 623 toddlers with a second Bayley test developed at least one ARI before their second test (cumulative 1238 ARIs). Parents reported fever and/or feverishness for 555 (79%) of the 703 children with ARIs before their first Bayley and 421 (88%) of the 480 children with ARIs before their second Bayley. An additional 20 (5%) had non‐respiratory febrile illnesses before their first Bayley and 11 (8%) before their second Bayley. Six children had febrile seizure, all during infancy. Ninety‐one (9%) were hospitalized for ARI before their first Bayley and 51 (8%) before their second Bayley. Respiratory samples were obtained for 526 (67%) of the 780 children with at least one ARI; 227 (43%) tested positive for rhinovirus, 142 (27%) tested positive for RSV, 63 (12%) for human metapneumovirus, 58 (11%) for influenza viruses, 39 (7%) for adenoviruses, 26 (5%) for parainfluenza virus 1, 9 (2%) for parainfluenza virus 2, and 55 (10%) for parainfluenza virus 3.

### 3.5 Bayley III results

We tested the development of 1062 infants a mean 368 days (IQR 366–376 days) after birth and retested 623 (57%) infants a mean 733 (IQR 730–739) days after birth (Table 3); only 36 (5.8%) had a second Bayley <6 months after the first. In Panama, children scored a mean 107 points (standard deviation [SD] 16) in cognitive development compared with 118 (SD = 13) in El Salvador during their first year of life (*p* < 0.0001). Similarly, infants in Panama scored 101 points (SD = 11) in motor and 97 points (SD = 7) in language development compared with 114 points (SD = 15) and 107 points (SD = 9) in El Salvador during their first year of life (*p* < 0.0001). Participants scored a mean of 102 points (SD = 12) in cognitive, 106 points (SD = 12) in motor, and 95 points (SD = 8) in language development in their second Bayley.

**TABLE 3:** First and second Bayley scores by maternal and child characteristics

First Bayley scores mean (standard deviation), n = 1062							Second Bayley scores, n = 623					
Parameter	Cognitive	*p* ^a^	Language	*p* ^a^	Motor	*p* ^a^	Cognitive	*p* ^b^	Language	*p* ^b^	Motor	*p* ^b^
Panama	107 (16)	<0.001	97 (7)	<0.001	101 (11)	<0.001	100 (14)	<0.001	91 (8)	<0.001	102 (12)	<0.001
El Salvador	118 (13)		107 (9)		114 (15)		104 (11)		97 (8)		109 (11)	
Before first Bayley							Before second Bayley					
Any ARI	112 (16)	0.00	102 (10)	0.21	108 (15)	0.84	102 (13)	0.18	95 (9)	0.08	107 (12)	0.02
	117 (13)		105 (10)		110 (15)		102 (11)		95 (8)		105 (11)	
Any fever	112 (15)	0.02	102 (10)	0.44	108 (15)	0.54	102 (13)	0.45	95 (9)	0.01	107 (13)	0.02
	116 (16)		105 (9)		111 (16)		102 (11)		94 (7)		106 (11)	
Sought hospital for ARI	111 (14)	0.75	101 (9)	0.87	107 (15)	0.49	102 (16)	1.00	94 (10)	0.79	106 (15)	0.72
	113 (15)		102 (10)		108 (15)		102 (12)		95 (8)		107 (12)	
Influenza^c^	116 (15)	0.17	102 (9)	0.67	110 (15)	0.77	99 (9)	0.05	95 (8)	0.22	106 (12)	0.73
	115 (14)		104 (10)		109 (15)		103 (12)		94 (8)		106 (11)	
RSV^c^	113 (12)	0.41	102 (11)	0.52	109 (14)	0.56	104 (10)	0.12	94 (9)	0.59	109 (13)	0.07
	115 (13)		103 (10)		109 (16)		102 (12)		95 (8)		107 (12)	
Adenovirus^c^	114 (11)	0.67	104 (9)	0.37	107 (18)	0.49	100 (11)	0.28	92 (8)	0.07	104 (10)	0.19
	112 (13)		102 (10)		108 (15)		103 (13)		95 (9)		108 (13)	
HMPV^c^	114 (14)	0.71	104 (11)	0.78	109 (16)	0.41	101 (11)	0.53	94 (7)	0.22	110 (13)	0.56
	112 (13)		102 (10)		108 (15)		103 (14)		95 (9)		108 (13)	
Parainfluenza 1^c^	117 (12)	0.38	107 (13)	0.07	107 (18)	0.80	101 (11)	0.63	97 (12)	0.70	107 (15)	0.50
	112 (13)		102 (10)		108 (15)		102 (13)		95 (9)		108 (13)	
Parainfluenza 2^c^	113 (13)	0.07	112 (3)	0.43	116 (6)	0.91	110 (10)	0.33	99 (6)	0.82	113 (9)	0.82
	113 (13)		102 (10)		108 (15)		102 (13)		95 (9)		108 (13)	
Parainfluenza 3^c^	109 (14)	0.58	101 (10)	0.67	104 (14)	0.63	102 (14)	0.74	93 (9)	0.19	108 (12)	0.75
	113 (13)		103 (10)		109 (15)		102 (13)		95 (9)		108 (13)	
Birth characteristics												
Female	113 (15)	0.81	103 (10)	0.11	108 (15)	0.97	103 (11)	0.13	96 (8)	0.00	107 (12)	0.34
	113 (15)		102 (9)		108 (15)		101 (13)		93 (9)		106 (12)	
Twins	111 (12)	0.88	94 (10)	0.05	99 (8)	0.25	101 (11)	0.89	93 (5)	0.76	106 (10)	0.69
	113 (15)		102 (10)		108 (15)		102 (12)		95 (8)		107 (12)	
Premature	113 (14)	0.62	104 (11)	0.61	107 (16)	0.25	104 (13)	0.37	95 (8)	0.77	107 (12)	0.71
	113 (15)		102 (10)		108 (15)		102 (12)		95 (8)		106 (12)	
Weight ≤ −2 *Z*	114 (14)	0.99	101 (9)	0.21	106 (15)	0.33	102 (11)	0.88	94 (6)	0.52	110 (10)	0.63
	113 (14)		102 (10)		108 (15)		102 (13)		95 (8)		106 (12)	
Length ≤ −2 *Z*	112 (12)	0.18	103 (7)	0.72	108 (16)	0.49	105 (15)	0.33	95 (6)	0.75	111 (10)	0.55
	113 (14)		102 (10)		108 (15)		102 (12)		95 (9)		106 (12)	
Head circ. ≤ −2 *Z*	114 (14)	0.62	103 (10)	0.82	105 (15)	0.24	103 (12)	0.80	95 (6)	0.98	108 (12)	0.72
	113 (14)		102 (10)		6108 (15)		102 (13)		95 (9)		107 (12)	
Maternal characteristics												
Extreme age^d^	113 (16)	0.12	103 (10)	0.29	109 (16)	0.18	103 (10)	0.92	96 (8)	0.60	107 (11)	0.44
	113 (15)		102 (10)		108 (15)		102 (13)		94 (8)		106 (94)	
Primigravid	114 (15)	0.58	104 (10)	0.19	109 (15)	0.66	103 (11)	0.54	95 (7)	0.83	107 (11)	0.89
	113 (15)		101 (10)		107 (15)		102 (13)		94 (9)		106 (13)	
Indigenous	106 (13)	0.57	95 (8)	0.01	98 (14)	0.01	102 (13)	0.19	90 (9)	0.47	105 (14)	0.17
	114 (15)		103 (10)		109 (15)		102 (12)		95 (8)		107 (12)	
Married	116 (16)	0.68	106 (10)	0.04	111 (15)	0.35	103 (11)	0.89	97 (9)	0.15	111 (13)	0.00
	113 (15)		102 (10)		107 (15)		102 (12)		94 (8)		106 (12)	
≤Primary school	113 (14)	0.22	103 (10)	0.05	109 (16)	0.19	102 (13)	0.93	94 (9)	0.38	106 (13)	0.21
	113 (15)		102 (10)		108 (15)		102 (12)		95 (8)		107 (12)	
Intimate partner violence	112 (15)	0.95	102 (10)	0.75	107 (15)	0.06	102 (13)	0.43	94 (8)	0.91	107 (13)	0.72
	115 (16)		104 (10)		111 (15)		5102 (11)		95 (8)		105 (10)	
Pre‐existing condition	112 (17)	0.44	101 (8)	0.17	105 (16)	0.61	5100 (16)	0.27	94 (10)	0.60	104 (14)	0.91
	113 (15)		103 (10)		108 (15)		102 (12)		95 (8)		107 (12)	
Alcohol	112 (8)	0.25	100 (13)	0.15	104 (16)	0.58	102 (15)	0.96	92 (10)	0.66	102 (7)	0.64
	113 (15)		102 (10)		108 (15)		102 (12)		95 (8)		107 (12)	
Smoker	120 (11)	0.15	107 (8)	0.07	109 (13)	0.60	98 (6)	0.38	86 (9)	0.04	100 (7)	0.41
	113 (15)		102 (10)		108 (15)		102 (12)		95 (8)		107 (12)	
Pregnancy ARI	117 (13)	0.17	105 (10)	0.68	112 (17)	0.32	104 (12)	0.57	95 (8)	0.70	108 (11)	0.99
	113 (15)		102 (10)		108 (15)		102 (12)		95 (8)		106 (12)	
Influenza^c^	119 (10)	0.15	110 (8)	0.37	118 (16)	0.10	99 (8)	0.03	96 (4)	0.32	101 (4)	0.00
	115 (13)		105 (12)		112 (3)		113 (4)		99 (11)		126 (27)	
Zika^e^	117 (13)	0.34	101 (9)	0.24	105 (11)	0.57	113 (11)	0.79	103 (9)	0.24	111 (7)	0.96
	113 (13)		102 (9)		105 (13)		111 (13)		98 (9)		111 (12)	

Abbreviations: ARI, acute respiratory illnesses; CDC, Centers for Disease Control and Prevention; rtPCR, real‐time reverse transcription polymerase chain reaction.

^a^ *p* values determined by univariate multilevel models.

^b^ *p* values determined by bivariate multilevel models, controlling for first cognitive score

^c^ Detection of viral ribonucleic acid through molecular rtPCR testing using CDC protocols at any time before the first Bayley (panel on the left) or any time before second Bayley (panel on the right).

^d^ Extreme maternal age defined as <18 or >35 years.

^e^ 432 (41%) of 1065 mothers participated in Zika substudy.

### 3.6 Missing data

Thirty‐two percent (502) of the 1567 enrolled neonates were lost to follow‐up during their first year of life, and an additional 458 (29%) were lost to follow‐up in the second year. Most losses to follow‐up were because the child missed more than two clinic visits. Factors measured at baseline that were associated (*p* > 0.05) with increased odds of dropout were living in Panama, White, or Indigenous race/ethnicity and mothers reporting a pre‐existing condition. The average number of children in the families of children who dropped out was higher than that for children who did not drop out (mean 2.2 vs. 1.1, *p* < 0.01). Factors with decreased odds of dropping out were Mestizo race/ethnicity, first pregnancy, and being poor.

### 3.7 Associations between ARIs, fever, influenza, and cognitive scores

In the first year of life, no maternal factors were associated with lower Bayley cognitive scores (Table 3). Children who ever experienced ARI scored an average of 2.6 points lower on their cognitive scores than children without any reported ARIs (*p* = 0.004), and children who experienced fever scored an average of 2.1 points lower compared with children who had not reported fever (*p* = 0.015). Multivariate multilevel models indicated that children who reported any ARI scored 3.0 points lower than children who did not report any ARIs (*p* = 0.001), controlling for any maternal respiratory illness, maternal Zika illness, infant flu vaccination, being born during an influenza epidemic, and the number of children in the household. Dropping twins from the analysis did not change the results of the multivariate analysis.

In the second year of life, maternal factors associated with Bayley cognitive scores were maternal influenza vaccination (*p* = 0.071) and toxoplasmosis (*p* = 0.058), controlling for the first Bayley score. Other factors associated with the second Bayley score were being poor (*p* = 0.014), reporting influenza in the second year of life (0.045) and size of household (*p* = 0.08), controlling for the first Bayley score. Multivariate multilevel models indicated that children having a laboratory‐confirmed positive influenza test at any time before their second Bayley test scored 4.0 points lower on the Bayley cognitive test compared with children who did not have influenza (*p* = 0.042), controlling for influenza vaccination status, any maternal respiratory illness, maternal Zika infection, being born during the influenza season, the number of children under 5 years in the household, and the first Bayley cognitive score. Children with respiratory syncytial virus, parainfluenza viruses 1–3, adenoviruses, human metapneumovirus, and rhinovirus had similar cognitive scores to children who tested negative for these viruses in multivariate analysis. Dropping twins did not change the results of the multivariate analysis.

## 4 DISCUSSION

ARI and fever were associated with lower cognitive scores among children aged 12 months. The clinical significance of these lower cognitive scores is unclear and will be studied in a subsequent phase of the cohort. It is also unclear why ARI and fever were associated with lower cognitive scores, but it is possible that this finding might have been associated with increased proinflammatory interleukins levels during illness.^12^ For example, it is possible that cognitive function among children with ARIs might have been affected by proinflammatory interleukins precipitating subsequent brain remodeling, as suggested by animal models infected with influenza viruses.^9^, ^10^ One might also speculate that ARIs led to Eustachian tube dysfunction, serous otitis, or other upper respiratory tract sequela that affected audition; while we did not perform audiometry, children with ARIs that occurred days before Bayley testing had similar cognitive scores to those with ARIs that occurred months before, when effects of upper respiratory tract dysfunction might have dissipated among most children.

Children with laboratory‐confirmed influenza illnesses, at any time before their second Bayley, also scored lower in their second‐year cognitive tests than children without influenza; these differences were statistically significant when controlling for the first cognitive test score and ARIs. Influenza triggers a unique cytokine response among respiratory viruses,^21^ often manifests with fever among children,^22^ and sporadically leads to encephalitis.^23^ If the impact of influenza illness on cognition is replicable and lasts beyond the second year of life, this might increase the value of influenza vaccination to protect childhood development. Evidence that influenza might have a lasting impact on cognition might fundamentally change the value proposition of influenza vaccines and therapeutics. In the absence of such evidence, having influenza among cohort children alone reaffirms the value of Panama and El Salvador's policy to fully immunize children against influenza.

Most children had febrile ARIs by their second birthday. It is therefore unclear whether febrile ARIs would have affected cognition beyond age 24 months among children already performing poorly on cognitive tests or if such childhood illnesses might later manifest in poor scholastic achievement, as suggested by a recent Danish cohort of half a million children.^24^ Such studies suggest the potential benefit of prevention and treatment febrile illnesses during infancy. Most febrile ARIs were caused by laboratory‐confirmed respiratory viruses; influenza specifically caused approximately 1/9 of detected febrile ARIs among cohort children. While vaccines, monoclonal antibodies, and antivirals are still in development for most respiratory viruses, Panama and El Salvador purchase vaccines and antivirals for the prevention and treatment of influenza. Despite progressive policies and government investment in free vaccination, only 17% cohort infants were fully vaccinated against influenza and none were treated with antivirals. Unvaccinated and partially vaccinated influenza‐naïve children would have been at higher risk of developing influenza illnesses than fully vaccinated children,^25^ and children untreated with antivirals would have been at higher risk of severe complications from influenza.^15^, ^26^ Influenza vaccines post introduction evaluations,^27^ knowledge attitude and practice studies (e.g., of mothers and pediatricians) to explore how to improve influenza vaccine coverage prior to the influenza season,^28^ and empiric antiviral treatment during the season might optimize investments in influenza prevention and control.

We identified maternal and childhood risk factors commonly associated with lower cognition,^13^ but after accounting for country of origin, maternal and childhood risk factors, and/or baseline Bayley scores, only ARIs, fevers, and/or influenza illnesses were significantly associated with Bayley scores. Other household factors such as having mothers with elementary school education or less, negatively affected scores, but these differences did not remain significant in multivariate analyses. It is therefore possible that some associations were masked by ARI case status and difficult to independently identify because of limited sample size.

Our study had strengths and limitations. Unlike previous cohorts, we carefully followed mothers during pregnancy, recorded gestational age using ultrasound, and swabbed ARIs. We then followed children for 2 rather than 1 year and documented findings following STROBE guidelines.^12^ However, our study had important limitations. First, we had unexpectedly high losses to follow‐up from enrollment of a very mobile population, missed swabbing opportunities, and potentially misclassified influenza case status because of passive follow‐up and an insensitive ARI case definition for testing.^22^ Several baseline variables were associated with loss to follow‐up, notably Mestizo race/ethnicity and poor economic status were associated with study retention; these variables were also associated with first or second cognitive scores in univariate/bivariate models and excluded from multivariate models. These variables will be important to test in future studies. While Bayley III scores were internally consistent (coefficients ranged from 0.86 to 0.91), it is possible that the instrument imperfectly assessed cognitive development among some racial and/or ethnic minority^29^ children or whose households were bilingual.^30^ Although we initially planned to obtain blood work to document proinflammatory marker levels,^12^ we did not have the resources to do so or to systematically seek to identify encephalitis, electroencephalograms, and audiology assessments from ill children to explore mechanisms of action and identify ARI sequelae.^23^ We also did not have the resources to systematically document neurodevelopmental diagnoses, maternal depression, postnatal exposure to violence, home stimulation, micromalnutrition, toxicants, concomitant illnesses, or visual and/or auditory impairment^31^ that might have been attributed to lower cognitive scores but hope to do so in our future studies. Last, while our findings seem consistent with those of our cohorts in Asia, our results may not be generalizable to other populations.

## 5 CONCLUSION

ARIs and fevers during infancy were associated with lower Bayley cognitive scores. Children with laboratory‐confirmed influenza illnesses during the first year of life scored lower in their second cognitive tests than those without influenza, but this difference was not statistically significant. Children who had laboratory‐confirmed influenza before their second year of life, however, had significantly lower cognitive scores when controlling for ARIs and country. It is unclear if children with lower cognitive scores catch up to their peers after 24 months of age. Larger and longer duration cohorts might be warranted to further explore associations between influenza and cognitive development, identify lasting impact beyond the age of 2 years, explore mechanisms of action, and determine the potential value of vaccines, antipyretics, and antiviral treatments in mitigation. Panama and El Salvador might benefit from program evaluations to optimize vaccination coverage and empiric ARI treatment with antivirals among very young children.

## AUTHOR CONTRIBUTIONS

**Eduardo Azziz‐Baumgartner:** Conceptualization; data curation; formal analysis; funding acquisition; methodology; resources; supervision; validation; visualization. **Rosalba Gonzalez:** Conceptualization; funding acquisition; investigation; methodology; project administration; supervision. **William Davis:** Data curation; formal analysis; methodology. **Arlene Calvo:** Data curation; investigation; project administration; supervision; validation. **Natalie Olson:** Data curation; formal analysis; validation; visualization. **Lauren Grant:** Data curation; formal analysis; methodology; validation. **Morgan Hess‐Holtz:** Data curation; project administration; resources; software; validation. **Vic Veguilla:** Data curation; funding acquisition; project administration; resources; supervision; validation. **Rafael Rauda:** Data curation; investigation; project administration; resources; supervision; validation. **Susan Cornelia Kaydos‐Daniels:** Conceptualization; funding acquisition; investigation; methodology; project administration; resources; software; supervision; validation. **Nestor Sosa:** Conceptualization; funding acquisition; project administration; resources; supervision. **Evelina I. Aedo Ruíz:** Data curation; investigation; methodology; project administration; resources; supervision; validation. **Julio Armero Guardado:** Investigation; project administration; resources; supervision; validation. **Rachael Porter:** Data curation; formal analysis; validation. **Danilo Franco:** Data curation; investigation; project administration; validation. **Juan Miguel Pascale:** Investigation; project administration; supervision; validation. **Georgina Peacock:** Supervision; validation.

## Data Availability

Data are available on request from the authors.

## References

[1] Peasah S , Azziz‐Baumgartner E , Breese J , Meltzer M , Widdowson M . Influenza cost and cost‐effectiveness studies globally—a review. Vaccine. 2013;31(46):5339‐5348. 10.1016/j.vaccine.2013.09.013 24055351

[2] Akins PT , Belko J , Uyeki TM , Axelrod Y , Lee KK , Silverthorn JJNC . H1N1 encephalitis with malignant edema and review of neurologic complications from influenza. Neurocrit Care. 2010;13(3):396‐406. 10.1007/s12028-010-9436-0 20811962 PMC7100075

[3] Glaser CA , Winter K , DuBray K , et al. A population‐based study of neurologic manifestations of severe influenza A(H1N1)pdm09 in California. Clin Infect Dis. 2012;55(4):514‐520. 10.1093/cid/cis454 22573853

[4] Goenka A , Michael BD , Ledger E , et al. Neurological manifestations of influenza infection in children and adults: results of a National British Surveillance Study. Clin Infect Dis. 2013;58(6):775‐784. 10.1093/cid/cit922 24352349

[5] Chaves SS . Neurologic complications of influenza in children. MJH Life Sci. 2013;30(8):26‐37.

[6] Chiu SS , Tse CYC , Lau YL , Peiris M . Influenza A infection is an important cause of febrile seizures. Pediatrics. 2001;108(4):e63‐e63. 10.1542/peds.108.4.e63 11581471

[7] Toovey S . Influenza‐associated central nervous system dysfunction: A literature review. Travel Med Infect Dis. 2008;6(3):114‐124. 10.1016/j.tmaid.2008.03.003 18486065

[8] Kuiken T , Taubenberger JK . Pathology of human influenza revisited. Vaccine. 2008;26:D59‐D66. 10.1016/j.vaccine.2008.07.025 19230162 PMC2605683

[9] Hosseini S , Wilk E , Michaelsen‐Preusse K , et al. Long‐term neuroinflammation induced by influenza A virus infection and the impact on hippocampal neuron morphology and function. J Neurosci. 2018;38(12):3060‐3080. 10.1523/JNEUROSCI.1740-17.2018 29487124 PMC6596076

[10] Kim JH , Yu JE , Chang B‐J , Nahm S‐S . Neonatal influenza virus infection affects myelination in influenza‐recovered mouse brain. J Vet Sci. 2018;19(6):750‐758. 10.4142/jvs.2018.19.6.750 30173495 PMC6265592

[11] Meisels SJ , Plunkett JW , Pasick PL , Stiefel GS , Roloff DW . Effects of severity and chronicity of respiratory illness on the cognitive development of preterm infants. J Pediatr Psychol. 1987;12(1):117‐132. 10.1093/jpepsy/12.1.117 3572673

[12] Jiang NM , Tofail F , Moonah SN , et al. Febrile illness and pro‐inflammatory cytokines are associated with lower neurodevelopmental scores in Bangladeshi infants living in poverty. BMC Pediatr. 2014;14(1):1‐9. 24548288 10.1186/1471-2431-14-50 PMC3936797

[13] Walker SP , Wachs TD , Grantham‐McGregor S , et al. Inequality in early childhood: risk and protective factors for early child development. Lancet. 2011;378(9799):1325‐1338. 10.1016/S0140-6736(11)60555-2 21944375

[14] Palache A , Oriol‐Mathieu V , Fino M , Xydia‐Charmanta M . Seasonal influenza vaccine dose distribution in 195 countries (2004–2013): Little progress in estimated global vaccination coverage. Vaccine. 2015;33(42):5598‐5605. 10.1016/j.vaccine.2015.08.082 26368399

[15] Dawood F , Jara J , Gonzalez R , et al. A randomized, double‐blind, placebo‐controlled trial evaluating the safety of early oseltamivir treatment among children 0‐9 years of age hospitalized with influenza in El Salvador and Panama. Antiviral Res. 2016;133:85‐94. 10.1016/j.antiviral.2016.07.007 27451343

[16] Palekar RS , Rolfes MA , Arriola CS , et al. Burden of influenza‐associated respiratory hospitalizations in the Americas, 2010‐2015. PLoS ONE. 2019;14(9):e0221479. 10.1371/journal.pone.0221479 31490961 PMC6730873

[17] WHO . Global Epidemiological Surveillance Standards for Influenza. Secondary Global Epidemiological Surveillance Standards for Influenza 2013. https://www.who.int/influenza/resources/documents/WHO_Epidemiological_Influenza_Surveillance_Standards_2014.pdf?ua=1

[18] Armstrong K , Agazzi HC , Aylward GP , Oakland T , Weiss LG . The Bayley‐III Cognitive Scale. In: Bayley‐III Clinical Use and Interpretation. Academic Press; 2010.

[19] Pearson . Bayley Scales of Infant and Toddler Development|Third Edition, 2021. https://www.pearsonassessments.com/store/usassessments/en/Store/Professional‐Assessments/Behavior/Adaptive/Bayley‐Scales‐of‐Infant‐and‐Toddler‐Development‐%7C‐Third‐Edition/p/100000123.html 10.1136/archdischild-2020-319063 32859738

[20] Durand LO , Cheng PY , Palekar R , et al. Timing of influenza epidemics and vaccines in the American tropics, 2002‐2008, 2011‐2014. Influenza Other Respi Viruses. 2016;10(3):170‐175. 10.1111/irv.12371 PMC4814866 26701079

[21] Melendi GA , Laham FR , Monsalvo AC , et al. Cytokine profiles in the respiratory tract during primary infection with human metapneumovirus, respiratory syncytial virus, or influenza virus in infants. Pediatrics. 2007;120(2):e410‐e415. 10.1542/peds.2006-3283 17671045

[22] Thompson M , Levine M , Bino S , et al. Underdetection of laboratory‐confirmed influenza‐associated hospital admissions among infants: a multicentre, prospective study. Lancet Child Adolesc Health. 2019;3(11):781‐794. 31492594 10.1016/S2352-4642(19)30246-9 PMC7029431

[23] Fowler Å , Stödberg T , Eriksson M , Wickström R . Childhood encephalitis in Sweden: Etiology, clinical presentation and outcome. Eur J Paediatr Neurol. 2016;12(6):484‐490. 10.1016/j.ejpn.2007.12.009 18313340

[24] Köhler‐Forsberg O , Sørensen H , Nordentoft M , McGrath J , Benros M , Petersen L . Childhood infections and subsequent school achievement among 598,553 Danish children. Pediatr Infect Dis J. 2018;37(8):731‐737. 10.1097/INF.0000000000001869 29278614

[25] El Omeiri N , Azziz‐Baumgartner E , Thompson MG , et al. Seasonal influenza vaccine effectiveness against laboratory‐confirmed influenza hospitalizations: first multi‐country estimates from Latin America. Vaccine. 2018;36(24):3555‐3566. 10.1016/j.vaccine.2017.06.036 28648543 PMC5988548

[26] Muthuri S , Venkatesan S , Myles P , et al. Effectiveness of neuraminidase inhibitors in reducing mortality in patients admitted to hospital with influenza A H1N1pdm09 virus infection: a meta‐analysis of individual participant data. Lancet. 2014;2(5):395‐404. 10.1016/S2213-2600(14)70041-4 PMC6637757 24815805

[27] WHO . Influenza Vaccine Post‐Introduction Evaluation (IPIE). 2020

[28] Ropero‐Álvarez A , El Omeiri N , Kurtis H , Danovaro‐Holliday M , Ruiz‐Matus C . Influenza vaccination in the Americas: progress and challenges after the 2009 A(H1N1) influenza pandemic. Hum Vaccin Immunother. 2016;12(8):2206‐2214. 27196006 10.1080/21645515.2016.1157240 PMC4994725

[29] Freeman Duncan A , Watterberg KL , Nolen TL , et al. Effect of ethnicity and race on cognitive and language testing at age 18‐22 months in extremely preterm infants. J Pediatr. 2012;160(6):966‐971. 10.1016/j.jpeds.2011.12.009 22269248 PMC3343209

[30] Thompson L , Peñaloza RA , Stormfields K , et al. Validation and adaptation of rapid neurodevelopmental assessment instrument for infants in Guatemala. Child Care Health Dev. 2015;41(6):1131‐1139. 10.1111/cch.12279 26250756 PMC4715612

[31] MAL‐ED Network Investigators . Early childhood cognitive development is affected by interactions among illness, diet, enteropathogens and the home environment: findings from the MAL‐ED birth cohort study. BMJ Glob Health. 2018;3:e000752. 10.1136/bmjgh-2018-000752 PMC6058175 30058645

